# Supplementary material for: Comparison of methods for determining parameters related to ankle joint quasi-stiffness during quiet standing
Source: PLoS One. 2026 Apr 13;21(4):e0338581. doi: 10.1371/journal.pone.0338581 (PMC13075674; doi:10.1371/journal.pone.0338581)
Supplement: Attachment — Submitted filename: ResponseLetter-Final.pdf [file pone.0338581.s002.pdf]

Response to Reviewer #1:

We sincerely thank the reviewer for the positive evaluation of our study and for the constructive suggestions that helped us improve the clarity of the manuscript.

1) This study was aimed to investigate the relationship across 1, 2, and in healthy young individuals. The results showed that the good and moderate reliability between 1 and 2 suggests that both measures capture similar stiffness attributes relating to their neuro-mechanical components. Additionally, relatively high correlations of with 1 and with 2 suggest that the stochastic characteristics of center of pressure in the short period indeed reflect the overall quasi-stiffness at the ankle joint. Overall, the study is interesting, however there are some clarifications needed.

Thank you for the positive overall comment. In the following, we provided our comments to each point. All corresponding revisions are highlighted in red in the revised manuscript.

2) Comment#1: Introduction: please state hypothesis/hypotheses for this study.

We appreciate this helpful suggestion. We have revised the final paragraph of the Introduction to clearly and explicitly state the study hypotheses (Line 67-74). Specifically, we now indicate that we hypothesized (i) good reliability and strong correlation between K_qs1 and K_qs2, and (ii) a negative association between H_s and the quasi-stiffness measures.

3) Comment#2: Abstract, please insert effect size for repeated measure ANOVA performed.

Thank you for this important suggestion. We have now included the effect sizes (partial η^2) for the repeated-measures ANOVA in both the Abstract and the Results section to provide a more complete statistical reporting of our findings (Line 9-12, 149-151).

Response to Reviewer #2:

We sincerely thank the reviewer for the positive evaluation of our study and for the constructive suggestions that helped us improve the clarity of the manuscript.

1) Your article titled "Comparison of methods for determining parameters related to ankle joint quasi-stiffness during quiet standing" is a valuable contribution to the field of biomechanics and postural control because of the unique perspective it offers. To make the scientific impact of your work as strong as possible, I suggest the following improvements.

Thank you for the positive overall comment. In the following, we provided our comments to each point. All corresponding revisions are highlighted in red in the revised manuscript.

2) First, it would be useful to have a deeper discussion about the mechanical and modeling reasons for the systematic difference (bias) found between the methods, using current literature to explain these findings.

We agree that a clearer mechanistic explanation of the systematic difference between K_qs1 and K_qs2 strengthens the interpretation of our findings.

In the revised manuscript (Discussion, "Relation between K_qs1 and K_qs2", Line 200-219), we have expanded the discussion to clarify the modeling and mechanical distinctions between the two approaches. Specifically, we now emphasize that K_qs2 is estimated from the local torque–angle slope at equilibrium during individual unit sways, thereby reflecting stiffness at specific transition points within the sway cycle. In contrast, K_qs1 is inferred from frequency-domain identification of the inverted pendulum model using the entire time series, representing the effective restoring stiffness governing the overall oscillatory behavior of the system. If the torque–angle relationship varies throughout the sway cycle, stiffness estimated from localized equilibrium segments may differ from stiffness inferred from the global oscillatory dynamics. This difference in temporal scale and signal utilization may contribute to the consistently larger magnitude of K_qs1 compared to K_qs2.

3) Second, I believe the limitations section needs to be more transparent about how the sample size affects the statistical power of your results. Since the study is limited to 11 healthy young male participants, it is difficult to apply these findings to other groups, and the lack of female participants and the narrow age range are significant weaknesses.

We thank the reviewer for this important and constructive comment. We agree that the relatively small sample size and homogeneous participant characteristics limit both statistical power and generalizability.

In the revised manuscript, we have expanded the Study Limitation section to more explicitly address these concerns (Line 247-256). Specifically, we clarify that although the sample size was relatively small ($n = 11$), the large correlations observed between H_s and the quasi-stiffness measures, as well as the moderate-to-large effect size for methodological differences (partial $\eta^2 = 0.42$), suggest that the primary findings are unlikely to be attributable to sampling variability alone. In contrast, the small effect sizes observed for eye condition ($\eta^2 = 0.06$) and interaction ($\eta^2 = 0.02$) indicate limited sensitivity to detect subtle effects, and these findings are therefore interpreted with appropriate caution.

We have also clarified that the exclusive inclusion of young healthy male participants restricts generalizability to females, older adults, and clinical populations.

We believe these revisions improve transparency regarding both statistical power and external validity while maintaining appropriate caution in interpretation.

4) Finally, adding a paragraph on how the relationship between the short-term scaling exponent and quasi-stiffness might look in different clinical groups, such as the elderly or people with neurological disorders, would greatly improve the clinical value and future research potential of your work.

We agree that discussing the potential behavior of the H_s–quasi-stiffness relationship in clinical populations strengthens the broader relevance of our findings.

In the revised manuscript (Discussion section, Line 233-244), we have expanded the final paragraph to explicitly address this point. Specifically, we now integrate prior evidence showing elevated H_s values in elderly and neurological populations (e.g., Parkinson's disease and stroke) with our present finding that higher H_s is associated with lower ankle quasi-stiffness. Based on this integration, we suggest that at-risk populations exhibiting elevated H_s may also demonstrate reduced ankle quasi-stiffness, although this relationship has not yet been directly verified.

We emphasize that future studies should concurrently quantify H_s and ankle quasi-stiffness in such populations to determine whether the inverse association observed in healthy young individuals is preserved or altered under pathological conditions. If confirmed, H_s, which is obtainable from force plate measurements alone, may serve as a clinically feasible surrogate biomarker of ankle quasi-stiffness regulation.
